# Fatty acid esters of azaspiracids identified in mussels (*Mytilus edulis*) using liquid chromatography-high resolution mass spectrometry

**DOI:** 10.1016/j.toxcx.2020.100059

**Published:** 2020-09-28

**Authors:** Elizabeth M. Mudge, Christopher O. Miles, William R. Hardstaff, Pearse McCarron

**Affiliations:** Biotoxin Metrology, National Research Council Canada, 1411 Oxford St., Halifax, NS, B3H 3Z1, Canada

**Keywords:** Azaspiracid, Fatty acid, Ester, LC-HRMS, Shellfish, Metabolism, Mussel

## Abstract

Azaspiracids (AZAs) are lipophilic polyether toxins produced by *Azadinium* and *Amphidoma* species of marine microalgae. The main dinoflagellate precursors AZA1 and AZA2 are metabolized by shellfish to produce an array of AZA analogues. Many marine toxins undergo fatty acid esterification in shellfish, therefore mussel tissues contaminated with AZAs were screened for intact fatty acid esters of AZAs using liquid chromatography-high resolution mass spectrometry. Acyl esters were primarily observed for AZAs containing hydroxy groups at C-3 with 3-*O*-palmitoylAZA4 identified as the most abundant acyl ester, while other fatty acid esters including 18:1, 16:1, 17:0, 20:2 and 18:0 acyl esters were detected. The structures of these acyl derivatives were determined through LC-MS/MS experiments, and supported by periodate cleavage reactions and semi-synthesis of palmitate esters of the AZAs. Esters of the hydroxy groups at C-20 or C-21 were not observed in mussel tissue. The relative proportion of the most abundant AZA ester was less than 3% of the sum of the major free AZA analogues. These findings reveal an additional metabolic pathway for AZAs in shellfish.

## Highlights

• Fatty acid esters of azaspiracids were identified in mussels *(Mytilus edulis*).
• Fatty acid esters of azaspiracids with hydroxy groups at C-3 were primarily observed.
• Fatty acid esters of regulated azaspiracids (AZA1, 2, −3) were absent.
• Structures were determined with LC-HRMS and confirmed by semi-synthesis of palmitate esters and periodate cleavage.
• This work reveals an additional metabolic pathway for azaspiracids in shellfish.

## 1 Introduction

**Fig. 1:**
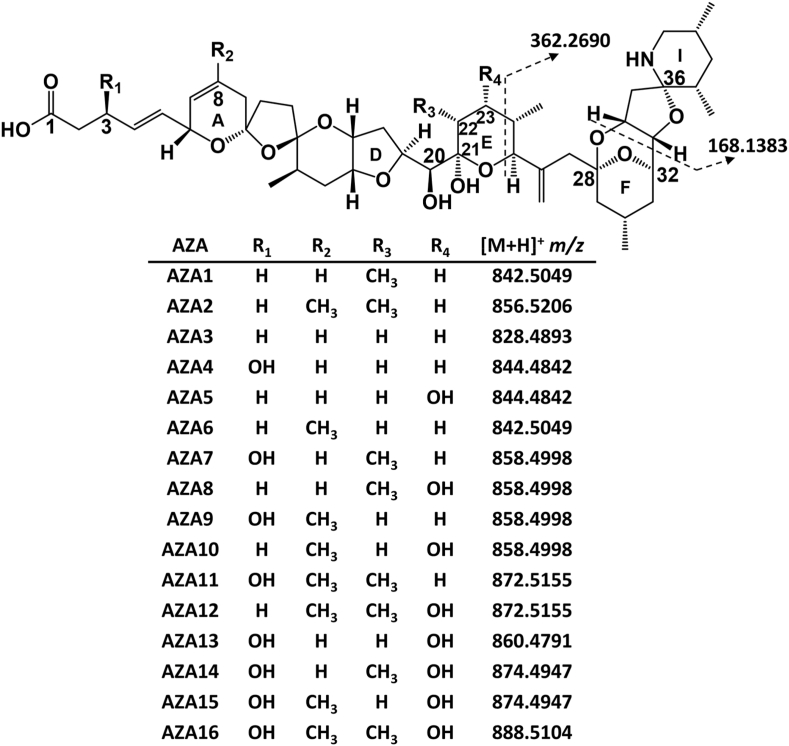
Structures of AZA1–16 and their protonated exact masses.

Since the first azaspiracid (AZA) poisoning event in 1995 from mussels (*Mytilus edulis*) harvested at Killary Harbour, Ireland, over 60 AZA analogues have been detected in shellfish and in *Amphidoma* and *Azadinium* spp. (Krock et al., 2019; Twiner et al., 2008). Azaspiracids have become a global concern, with *Azadinium* spp. and AZA-contaminated shellfish being detected in Europe, South America, North America, Australasia and Asia (Kim et al., 2017; Smith et al., 2016; Tillmann et al., 2009, 2016, 2017). AZA structures are characterized by three main functional groups: a cyclic amine, a tri-spiro assembly and a carboxylic acid. Two of the regulated analogues, AZA1 and -2, are produced by *A. spinosum*, while many others are shellfish metabolites and products from oxidation, hydroxylation, decarboxylation and dehydration (Fig. 1) (Hess et al., 2014; Kilcoyne et al., 2015a, 2018; McCarron et al., 2009; Tillmann et al., 2009).

These lipophilic toxins accumulate in shellfish tissues and in addition to the mechanisms described above have the potential to be metabolized similarly to other lipophilic toxin classes. The documentation of fatty acid acyl esters of okadaic acid, dinophysistoxins, pectenotoxins, brevetoxins, spirolides, pinnatoxins and gymnodimines highlights the potential for acylation of hydroxy groups to form esters and higher toxicity than indicated by the analysis of free toxins (Aasen et al., 2006; de la Iglesia et al., 2013; McCarron et al., 2012; Morohashi et al., 1995; Torgersen et al., 2008; Wilkins et al., 2006). The majority of AZAs in shellfish, especially those metabolized from AZA1 or -2, contain C-20 and C-21 hydroxy groups, while hydroxy groups can also be present at C-3 and C-23 (Fig. 1). The presence of these hydroxy groups presents the potential for acyl ester formation of AZAs in shellfish. The regulatory limit of 160 μg/kg AZA1 eq. set by the European Commission applies to free AZA1–3, which were the first AZAs identified in shellfish, while other analogues are generally observed at lower concentrations (European Commission, 2011; Hess et al., 2014). To date, *in vitro* potencies are reported as AZA2 > AZA6 > AZA34 ≈ 37-*epi*-AZA1 > AZA8 ≈ AZA3 > AZA1 > AZA4 ≈ AZA9 > AZA5 ≈ AZA10 > AZA33 > AZA26 (Kilcoyne et al., 2014a, 2014b, 2015b, 2018; Krock et al., 2015; Twiner et al., 2012).

AZA fatty acid esters were not detected in previous studies of mussel tissues (Rehmann et al., 2008). However, synthetic acylation reactions have shown that AZA1 can undergo fatty acid esterification (de la Iglesia et al., 2014). We herein report the use of liquid chromatography-high resolution mass spectrometry (LC-HRMS) for the detection of AZA fatty acid esters in mussel (*M. edulis*) tissues. The backbone AZA structures of these esters were verified based on product ion spectra, periodate cleavage experiments, and through comparison to semi-synthetic AZA acyl esters.

## 2 Materials and methods

### 2.1 Chemicals and reagents

LC-MS grade acetonitrile, methanol, ethanol, formic acid (~98%) and reagent grade ethyl acetate and hexane were from Fisher Scientific (Ottawa, ON, Canada). MS grade ammonium formate (>99%), HPLC grade pyridine (99.9%), reagent grade sodium periodate (>99%), 4-dimethylaminopyridine (DMAP, >98%), palmitic anhydride (99%) and sodium chloride were from Millipore-Sigma (Oakville, ON, Canada). Distilled water was ultra-purified to 18.2 MΩ cm using a Milli-Q water purification system (Millipore-Sigma, Billerica, MA, USA).

AZA1 calibration solution and mussel (*M. edulis*) matrix certified reference materials (CRM-AZA1b, CRM-AZA-Mus and CRM-FDMT1) were from the National Research Council Canada (Halifax, NS, Canada). In-house reference materials for AZA4, -7 and -9 were prepared as described previously (Kilcoyne et al., 2015). Mussel (*M. edulis*) tissues contaminated with AZAs provided by the Marine Institute, Ireland, were collected from the following locations (harvest year): Killary Harbour (1995); Bruckless, Donegal Bay (2005); and Gouladoo, Bantry Bay (2008).

### 2.2 Sample preparation

The hepatopancreas (HP) tissue of mussels containing AZAs from Bruckless was dissected from a subset of the mussels, homogenized and stored at −20 °C. Whole mussel tissues from Killary Harbour and Gouladoo were homogenized and stored at −20 °C prior to analysis.

Homogenized tissue (2 g) was extracted with MeOH (4 mL) by vortex mixing for 1 min and centrifuged at 3950 *g* for 5 min. The supernatant was decanted into a 10 mL volumetric flask, the pellet was re-extracted with MeOH (4 mL), centrifuged as above, and the supernatants were combined in the volumetric flask and made to volume with MeOH. An aliquot of the extract was filtered (0.2 μm Teflon spin-filter; Millipore-Sigma) prior to analysis.

### 2.3 AZA ester fraction preparation

Dissected HP (150 g) from Bruckless mussels was freeze-dried to yield 40 g of dry tissue, which was extracted with EtOH (3 × 100 mL) in a Waring blender. The extract was filtered (Whatman no. 5 filter paper) and evaporated under vacuum at 35 °C. The residue was partitioned between EtOAc (150 mL) and 1.0 M NaCl (100 mL). The fractions from each step of the procedure were analyzed by LC-MS to verify the presence of AZAs and AZA-esters. The EtOAc fraction was evaporated under vacuum and the residue partitioned between hexane (50 mL) and 90% MeOH (50 mL). The methanolic fraction was evaporated under vacuum. The residue was applied to an open silica gel column (10 cm × 5.0 cm i.d.) packed with 10–40 μm silica gel (Millipore-Sigma) and sequentially eluted with hexane–EtOAc (9:1), EtOAc–MeOH (7:3 and 1:1), and MeOH (250 mL each). The 7:3 and 1:1 EtOAc–MeOH fractions were evaporated under vacuum separately, and dissolved in MeOH (10 mL) for analysis.

### 2.4 LC-HRMS

Analyses were performed on an Agilent 1200 LC equipped with a binary pump, temperature controlled autosampler and column compartment coupled to a Q Exactive HF Orbitrap mass spectrometer (Thermo Fischer Scientific, Waltham, MA, USA) with a heated electrospray ionization probe (HESI-II). The chromatographic separation used a C8 column (100 × 2.1, 1.9 μm Thermo Hypersil Gold; Thermo Fischer Scientific, Waltham, MA, USA) with gradient elution. The mobile phase was water (A) and 95% MeCN (B), both containing 50 mM formic acid and 2 mM ammonium formate. The elution gradient (0.25 mL/min) was: 0–5 min, 50–100% B; 5–20 min, 100% B; 20–20.1 min, 100–50% B; and 5 min re-equilibration at 50% B. The column and sample compartments were maintained at 20 °C and 10 °C, respectively. The injection volume was 3 μL. The MS was calibrated from *m*/*z* 74–1622 according to the manufacturer's specification using the positive Pierce LTQ Velos calibration solution (Thermo-Fisher Scientific). Full scan data were collected from *m*/*z* 650–1200 using positive ionization with a spray voltage of 3.0 kV. The sheath gas pressure was 35 psi and auxiliary gas flow was 10 (arbitrary units). The capillary temperature was 350 °C and the heater temperature was 300 °C. The MS resolution setting was 60 000 with an AGC target of 1 × 10^6^ and a maximum injection time of 200 ms.

Data-independent acquisition (DIA) was used to screen for AZA-esters. Fifteen mass windows of 39 Da spanned the range from *m*/*z* 650–1200 with stepped collision energies of 35 and 65 eV to collect product ion spectra from all ions within each window (Table S1). The MS resolution was set at 15 000 with an AGC target of 2 × 10^5^ and a maximum injection time of 50 ms with a loop count of 8.

Data-dependent acquisition (DDA) was used to collect MS/MS product ion scans of the five most abundant ions in the full scan acquisition at each cycle, with an inclusion list for candidate AZA-esters from the DIA acquisition. The mass range for full scan acquisition was *m*/*z* 950–1200 with a resolution setting of 60 000, an AGC target of 1 × 10^6^ and maximum injection time of 100 ms. Product ion scans were acquired with an isolation window of 1 *m*/*z* with stepped collision energies of 35 and 65 eV. The resolution was set to 30 000 with an AGC target of 1 × 10^5^ and a maximum injection time of 50 ms.

### 2.5 Semi-synthesis of PalmitoylAZAs

Palmitic acid (16:0) esters of AZA1, -4, -7 and -9 were prepared using a procedure modified from previous applications to other algal toxins (Aasen et al., 2006; McCarron et al., 2012). All glassware used was dried in a desiccator overnight prior to sample preparation. Approximately 100 pmol of the individual AZA in MeOH was evaporated under nitrogen gas and placed in a desiccator for 4 h to remove residual water. The residue was dissolved in 100 μL of dry pyridine containing 30 mM palmitic anhydride and 100 mM DMAP. The solution was mixed at room temperature and allowed to stand for 30 min. The pyridine was dried under nitrogen and reconstituted in 100 μL of MeOH prior to LC-HRMS analysis.

### 2.6 Treatment with sodium periodate

An aliquot (20 μL) of the semi-synthetic 3-*O*-palmitoylAZA4 in MeOH (20 μL) was mixed with sodium periodate (50 mM, 2 μL) in an HPLC vial insert. An aliquot (20 μL) of the Bruckless HP extract was mixed with 50 mM sodium periodate (2 μL) in another HPLC vial insert. These solutions were vortex-mixed for 30 s and analyzed within 2 h by LC-HRMS.

## 3 Results and discussion

### 3.1 Detection of AZA fatty acid esters

**Fig. 2:**
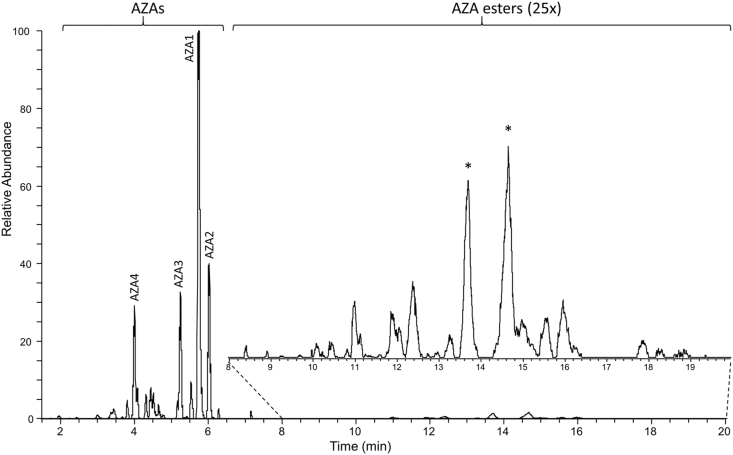
Sum of the extracted AZA diagnostic product ions at *m*/*z* 362.2690 and 168.1381 using a 5 ppm mass tolerance from the mass range of *m*/z 650 to 1200 collected using DIA acquisition of the HP extract of mussels collected from Bruckless. (*) 3-*O*-palmitoylAZA4 peaks.

Mussels collected from Bruckless, Ireland, in 2005 have previously been used for the isolation of AZA analogues, and for the production of reference materials (Hess et al., 2007; Kilcoyne et al., 2015a, 2015b; McCarron et al., 2015). Given that AZAs are sensitive to the base hydrolysis conditions typically used for indirect analysis of fatty acid esters (Alfonso et al., 2008), the Bruckless mussels were evaluated for intact fatty acid esters. To assist in the detection of potentially low levels of AZA esters, HP tissues were analyzed initially to provide a more highly concentrated sample for LC-HRMS analysis. After elution of known AZAs, the chromatographic separation was held at a high percentage organic mobile phase for 15 min to elute non-polar compounds. The samples were screened by LC-HRMS for AZA fatty acid esters using a DIA method which generated product ion scans from all ions within mass windows of width *m*/*z* 39, from *m*/*z* 650 to 1200, to determine the approximate mass range and potential precursor ions responsible for product ions characteristic of AZAs. The MS/MS fragmentation of AZAs has previously been evaluated in detail, where many AZA analogues have the same structural configuration towards the amino terminus, with characteristic product ions of *m*/*z* 362.2690 and 168.1383 resulting from fragmentation of the E and I rings, respectively (Fig. 1) (Rehmann et al., 2008). Given that there are no available hydroxy groups for known AZAs on this part of the molecule (C-24 to C-40), putative AZA fatty acid esters should also fragment to give these product ions. These two diagnostic ions were extracted from the DIA acquisition with a mass error of 5 ppm, which confirmed the known AZA analogues eluting in the first 7 min, while several later-eluting peaks also produced these characteristic AZA product ions (Fig. 2). These product ions from the later-eluting peaks were observed in the windows ranging from *m*/*z* 1052 to 1165.

**Fig. 3:**
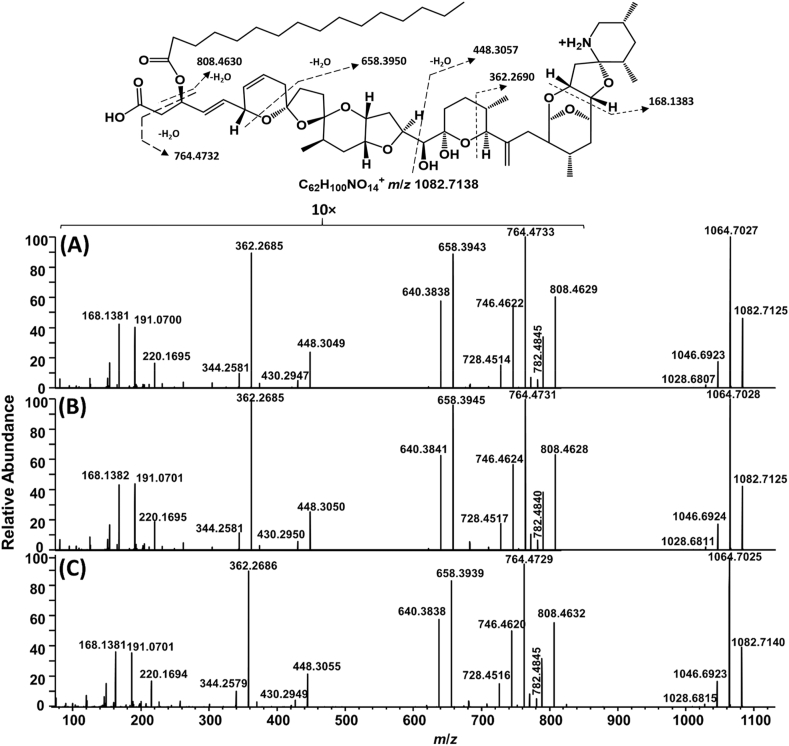
Extracted product ion spectra of (a,b) the two most abundant AZA-ester peaks using DDA acquisition in the fractionated HP of mussels collected in Bruckless. Both peaks arise from precursor ions at *m*/*z* 1082.7138 and the product ions are consistent with semi-synthetic 3-*O*-palmityolAZA4 (c) with a scheme showing the proposed origins of the major diagnostic ions with exact masses. The vertical scale from *m*/*z* 100–950 was expanded 10-fold to highlight diagnostic ions.

**Fig. 4:**
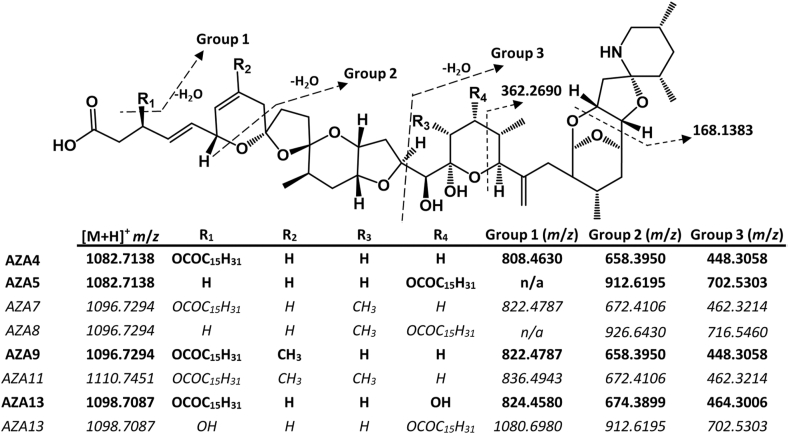
Potential palmitate (16:0) acyl esters of azaspiracids containing 3- and 23-hydroxy groups and the exact masses of their respective products ions (Groups 1–3) used to screen the HRMS data. Note that no esters of AZA7, -8 and -11 were detected in the HP tissue extract, and these entries are shown in italics.

To facilitate MS characterisation of these low-level AZA metabolites, an extract of the Bruckless mussel HP was fractionated and concentrated using silica gel chromatography (Kilcoyne et al., 2012). These fractions were used in targeted MS/MS experiments. The two most prominent peaks giving the diagnostic AZA product ions, eluting at 13.7 and 14.7 min (Fig. 2), had a pseudomolecular ion [M+H]^+^ of *m*/*z* 1082.7145, consistent with C_62_H_100_O_14_N^+^ (Δ 0.6 ppm). These two peaks had identical product ion spectra (Fig. 3a and b). As is typical for AZAs, there was a series of water losses from the pseudomolecular ion. The *m*/*z* 808.4629 product ion (C_46_H_66_O_11_N^+^, Δ −0.2 ppm) is consistent with neutral loss of palmitic acid (hexadecanoic) and one molecule of water, suggesting the originally metabolized AZA would have a [M+H]^+^ of *m*/*z* 844.4842 (C_46_H_70_O_13_N^+^) prior to forming a palmitate ester (16:0). The product ion at *m*/*z* 782.4845 (C_45_H_68_O_10_N^+^, Δ 0.9 ppm) is consistent with neutral loss of the fatty acid together with elimination of C-1 as CO_2_. Two ions that provided additional structural information on the AZA backbone were *m*/*z* 658.3943 (C_37_H_56_O_9_N^+^, Δ −1.0 ppm) arising from retro-Diels–Alder cleavage of the A-ring plus loss of water, and *m*/*z* 448.3049 (C_26_H_42_O_5_N^+^, Δ −1.9 ppm) from cleavage of C-19–C-20 with loss of water (Fig. 4). These product ions, in addition to the *m*/*z* 808.4630 ion, are characteristic of AZA4 (Rehmann et al., 2008), and strongly suggest that the two peaks arose from 3-*O*-palmitoylAZA4. The identical MS/MS fragmentation of these two peaks suggests that the location of acylation was the same, in addition to the backbone structure of this compound. The presence of two peaks suggests a variation in the fatty acid chain (e.g. branching) or epimerization in the AZA backbone that is not distinguishable by mass spectrometry, as has been observed in other fatty acid esters (McCarron et al., 2012; Torgersen et al., 2008).

The 3-*O*-palmitoylAZA4 was synthesized by acylation of AZA4 with palmitic anhydride. The acylation reaction was performed at room temperature where AZA4 reacted readily with palmitic anhydride. The AZA4 peak area was reduced by 80% after 30 min, producing two main products. The first product had an [M+H]^+^ at *m*/*z* 826.4743 (C_46_H_68_O_12_N^+^, Δ 0.8 ppm) (Fig. S1) corresponding to dehydration of AZA4, and a second later-eluting product with [M+H]^+^ at *m*/*z* 1082.7158 (C_62_H_100_O_14_N^+^, Δ 1.8 ppm) corresponding to 3-*O*-palmitoylAZA4. The retention time of the semi-synthetic AZA4 palmitic acid ester was identical to the later-eluting 3-*O*-palmitoylAZA4 peak in the Bruckless sample (Fig. S2). The Bruckless sample product ion spectra is consistent with the proposed structure and identical to that of the semi-synthetic compound (Fig. 3c), confirming that these peaks are acyl esters of AZAs. Based on confidence criteria outlined by Schymanski et al. (2014) the data acquired confirms the identity of 3-*O*-palmitoylAZA4 in the mussel tissues by establishing the highest level of confidence (level 1). The majority of the additionally identified AZA-esters described herein meet the requirements for level 2b confidence based on the presence of diagnostic fragments observed in the experimental data in comparison with this confirmed structure (Schymanski et al., 2014).

Previous quantitative analysis has shown that AZA4 is the most abundant of the non-regulated AZAs in the Bruckless HP (McCarron et al., 2015). Table 1 summarizes the observed AZA4 esters detected in the HP tissue based on exact mass and the presence of three diagnostic AZA4 fragments (*m*/*z* 808.4630, 658.3950 and 448.3058). The most significant were consistent with 16:0, 18:1, 16:1, 17:0, 20:2 and 18:0 3-*O*-acyl esters, based on peak area, which is similar to the fatty acid ester profiles observed for other acylated marine algal toxins in mussel tissues (Aasen et al., 2006; de la Iglesia et al., 2013; McCarron et al., 2012; Torgersen et al., 2008).

**Table 1:** Accurate masses (for [M+H]^+^), retention times and abundances (relative percentage to 3-*O*-palmitoylAZA4) of AZA4 and AZA9 fatty acid acyl esters in the Bruckless mussel HP tissue.

AZA	Fatty Acid	C:DB^a^	Retention Time (min)	Peak Area Relative to 3-*O*-palmitoylAZA4 (%)	Neutral Molecular Formula	Accurate Mass	Δ (ppm)
AZA4	Tetradecaenoic	14:1	10.0	1.8	C_60_H_93_O_14_N	1052.6671	0.2
	Tetradecanoic	14:0	12.0	8.1	C_60_H_95_O_14_N	1054.6846	2.0
	Pentadecanoic	15:0	13.2	5.7	C_61_H_97_O_14_N	1068.7003	2.0
	Hexadecatetraenoic	16:4	8.9	1.7	C_62_H_91_O_14_N	1074.6544	2.9
	Hexadecadienoic	16:2	9.5	5.7	C_62_H_95_O_14_N	1078.6840	1.4
	Hexadecaenoic	16:1	12.3	19.1	C_62_H_97_O_14_N	1080.7000	1.7
	Hexadecanoic^b^	16:0	14.7	100.0	C_62_H_99_O_14_N	1082.7159	1.9
	Heptadecaenoic	17:1	13.6	6.3	C_63_H_99_O_14_N	1094.7146	0.7
	Heptadecanoic^b^	17:0	15.9	12.3	C_63_H_101_O_14_N	1096.7318	2.1
	Octadecatetraenoic	18:4	9.9	3.1	C_64_H_95_O_14_N	1102.6862	3.3
	Octadecatrienoic	18:3	11.1	1.0	C_64_H_97_O_14_N	1104.7001	1.7
	Octadecadienoic	18:2	12.7	3.3	C_64_H_99_O_14_N	1106.7160	2.0
	Octadecaenoic^b^	18:1	15.6	20.2	C_64_H_101_O_14_N	1108.7315	1.8
	Octadecanoic^b^	18:0	17.9	9.8	C_64_H_103_O_14_N	1110.7471	1.8
	Eicosapentaenoic	20:5	10.4	3.0	C_66_H_97_O_14_N	1128.7006	2.1
	Eicosatetraenoic	20:4	11.8	0.7	C_66_H_99_O_14_N	1130.7136	−0.2
	Eicosadienoic	20:2	15.9	10.8	C_66_H_103_O_14_N	1134.7475	2.1
	Eicosenoic	20:1	18.9	5.9	C_66_H_105_O_14_N	1136.7626	1.6
	Docosahexaenoic	22:6	10.9	7.1	C_68_H_99_O_14_N	1154.7168	2.6
	Docosapentaenoic	22:5	12.4	2.0	C_68_H_101_O_14_N	1156.7310	1.3
	Docosadienoic	22:2	19.3	4.9	C_68_H_107_O_14_N	1162.7778	1.2
AZA9	Tetradecanoic	14:0	12.7	5.7	C_61_H_97_O_14_N	1068.6971	−1.0
	Pentadecanoic	15:0	14.0	1.9	C_62_H_99_O_14_N	1082.7153	1.4
	Hexadecaenoic	16:1	13.0	5.5	C_63_H_99_O_14_N	1094.7146	0.7
	Hexadecanoic^b^	16:0	15.5	7.6	C_63_H_101_O_14_N	1096.7314	1.7
	Heptadecanoic	17:0	17.0	0.5	C_64_H_103_O_14_N	1110.7446	−0.5
	Octadecaenoic^b^	18:1	16.5	2.2	C_65_H_103_O_14_N	1122.7440	−1.0
	Docosahexaenoic	22:6	11.5	1.1	C_69_H_101_O_14_N	1168.7315	1.7

Note: Retention times of free AZA4 and AZA9 were 3.92 and 4.22 min, respectively.

a Carbons:desaturation.

b Two peaks were observed with identical product ion spectra. RT is reported for the second peak, peak areas were combined for the two, Δ < 3 ppm for the first peak.

The regulated AZA1–3 analogues contain free hydroxy groups on C-20 and C-21 (Fig. 1), while AZA4 has an additional hydroxy group on C-3. Exact mass searches were negative for fatty acid esters of the regulated AZAs, such as the 16:0 acyl ester of AZA1 ([M+H]^+^ *m*/*z* 1080.7346), in the HP tissue, suggesting that the C-20 and C-21 hydroxyl groups are resistant to esterification with fatty acids in mussels, possibly due to steric hindrance. Several additional AZAs are hydroxylated at C-3, including AZA7, -9, -11, -13, while AZA5, -8 and -13 possess C-23 hydroxy groups that could also undergo fatty acid esterification.

To screen for the presence of other potential AZA esters in the Bruckless HP tissue, calculated MS/MS product ions for palmitate esters of the C-3 and C-23 hydroxylated AZAs were generated (Fig. 4) based on Rehmann et al. (2008) and used as a basis for identification. The fragments labelled as Groups 1–3 are diagnostic for the various potential AZA acyl esters. Fatty acid esters were observed for AZA9, with trace levels of AZA5 and AZA13 esters also detected. After AZA4, the AZA9 fatty acid esters were most abundant.

**Fig. 5:**
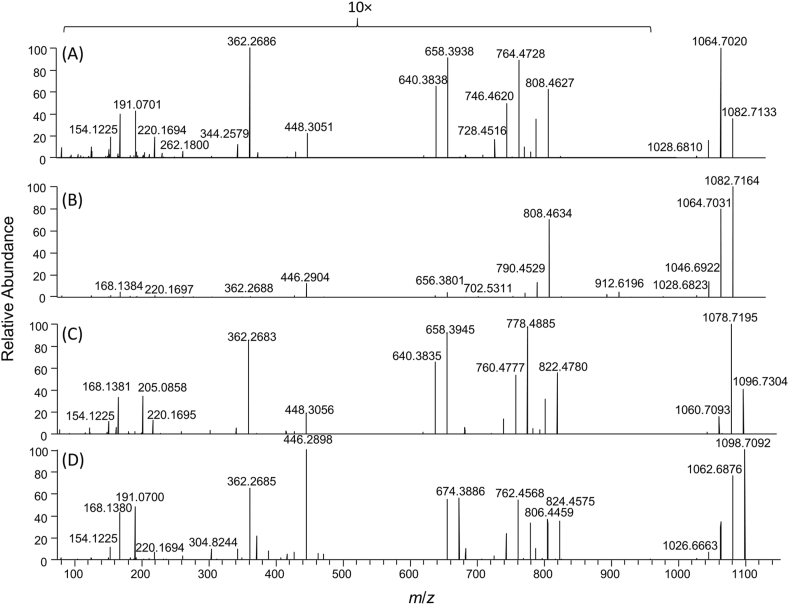
Product ion spectra of AZA acyl esters: (a) 3-*O*-palmitoylAZA4; (b) 23-*O*-palmitoylAZA5; (c) 3-*O*-palmitoylAZA9, and; (d) 3-*O*-palmitoylAZA13, obtained using DDA acquisition of the fractionated mussel hepatopancreas from Bruckless. The vertical scale from *m*/*z* 100–950 was expanded 10-fold to highlight diagnostic ions for (a), (c) and (d).

The diagnostic product ions *m*/*z* 822.4780 (C_47_H_68_O_11_N^+^, Δ −0.8 ppm), 658.3945 (C_37_H_56_O_9_N^+^, Δ −0.7 ppm) and 448.3056 (C_26_H_42_O_5_N^+^, Δ −0.3 ppm) were used for identification of the principal AZA9 fatty acid esters present (Table 1; Fig. 5c). The majority of the other esters observed were present in low abundance relative to those of AZA4. The most abundant acyl ester peak for each AZA in the HP tissue extract were the palmitate (16:0) esters. The mass spectra for 3-*O*-palmitoylAZA4, 23-*O*-palmitoylAZA5, 3-*O*-palmitoylAZA9 and 3-*O*-palmitoylAZA13 (Fig. 5) detected in the HP tissue each displayed the diagnostic ions depicted in Fig. 4.

A very low abundance peak with *m*/*z* 1082.7134 (C_62_H_100_O_14_N^+^, Δ −0.4 ppm) having product ions with *m*/*z* 912.6196 (C_53_H_86_O_11_N^+^, Δ 0.01 ppm) and *m*/*z* 702.5311 (C_42_H_72_O_7_N^+^, Δ 1.0 ppm) was consistent with palmitate esterification of the 23-hydroxy group of AZA5 (23-*O*-palmitoylAZA5; Fig. 5b). Product ions observed for 23-O-palmitoylAZA5 are consistent with fragmentation of the ester-free structure including *m*/*z* 446.2904 (C_26_H_40_O_5_N^+^, Δ 0.8 ppm) and *m*/*z* 656.3801 (C_37_H_54_O_9_N^+^, Δ 1.0 ppm) (Rehmann et al., 2008). This was the only AZA5 fatty acid ester observed in the HP tissues. Given the low relative abundance of this peak (ca 0.5% of 3-*O*-palmitoylAZA4), the metabolic pathway for esterification of AZA5, presumably for the 23-OH, appears to not be favored in the mussels. However, the lower relative abundance of AZA5 compared to AZA4 in these tissues is also a consideration. The concentrations of AZA5 and AZA9 are both about four times lower than AZA4 in the Bruckless tissue (McCarron et al., 2015) suggesting that 23-OH esterification in the case of AZA5 is disfavoured. Esters of AZA8 and -11 were not detected in the HP tissue nor in the concentrated fractions obtained from the silica gel fractionation. This suggests that the methyl group on C-22 may reduce the reactivity of 23-OH of AZAs toward fatty acids *in vivo*.

AZA13 contains hydroxy groups at both C-3 and C-23. 3-*O*-palmitoylAZA13 was observed at very low levels in the HP tissue, consistent with the low levels of free AZA13 in the source tissues with a pseudomolecular ion of *m*/z 1098.7102 (C_62_H_102_O_15_N^+^, Δ 1.3 ppm, Fig. 5d). This acyl ester is evident by the presence of the product ions at *m*/*z* 824.4575, 674.3886, and 464.3015 (C_46_H_66_O_12_N^+^, Δ −0.6 ppm, C_37_H_56_O_10_N^+^, Δ −1.9 ppm, and C_26_H_42_O_6_N^+^, Δ 1.8 ppm, respectively; Fig. 5d)). 23-*O*-palmitoylAZA13 was not confirmed in the mussel tissue in these experiments, further indicating that 23-OH esterification may not be favored.

Trace levels of 3-*O*-palmitoylAZA7 were detected in the 7:3 EtOAC:MeOH silica gel fraction, co-eluting with 3-*O*-margaroylAZA4 based on the observed product ions of *m*/*z* 822.4797, 672.4112 and 462.3218 (C_47_H_68_O_11_N^+^, Δ 1.2 ppm, C_38_H_58_O_9_N^+^, Δ 0.9 ppm, and C_27_H_44_O_5_N^+^, Δ 0.9 ppm, respectively; Fig. S3).

### 3.2 Further comparison with synthetic PalmitoylAZAs

The acylation reaction between palmitic anhydride and AZA9 resulted in a peak with identical retention time to the later-eluting of the two 3-*O*-palmitoylAZA9 peaks in the mussel HP tissue. The product ion spectrum (Fig. S4) was also identical with that observed for the same peak in the Bruckless sample, increasing the confidence level for the identification of 3-*O*-palmitoylAZA9. Acylation of AZA7 with palmitic anhydride also produced a peak with a product ion spectrum matching that expected for 3-*O*-palmitoylAZA7 (Fig. S5).

Previously, semi-synthetic preparation of 16:0 AZA1 was successful using three different acylation routes (de la Iglesia et al., 2014). The location of the fatty acid was not confirmed, but the authors suggested that it occurred at the hydroxy group at C-20 due to that location being less sterically hindered. Acylation of AZA1 with palmitic anhydride was performed in this work using a method similar to one used by de la Iglesia et al. (2014) except that the reaction was performed at room temperature rather than at 75 °C. The majority of AZA1 (approx. 70%) remained intact after reaction at room temperature (Fig. S6), but a small peak of palmitoylAZA1 was generated with an [M+H]^+^ at *m*/*z* 1080.7355 (C_63_H_102_O_13_N^+^, 0.9 Δ ppm) and retention time of 13.3 min, which was not present in the tissue extracts. The product ion spectrum confirmed acylation on the C-20 or C-21 hydroxy group via a characteristic product ion at *m*/*z* 928.6499 (C_54_H_90_O_11_N^+^, Δ −1.0 ppm) along with other AZA1 diagnostic product ions (Fig. S6). Given that fatty acid esters of AZAs without C-3 or C-23 hydroxy groups were not detected in mussels in this study, despite free AZA1–3 and −6 typically being among the major AZAs in the samples, suggests that the C-20 and C-21 hydroxy groups may be unreactive toward *in vivo* enzymatic acylation. This highlights the importance of comparing products of synthetic reactions with authentic naturally-incurred materials to verify their identities and biological significance.

### 3.3 Treatment of mussel extract and semi-synthetic 3-*O*-palmitoylAZA4 with NaIO_4_

Sodium periodate-mediated oxidative cleavage reactions provided additional structural information on the AZA esters. The 20,21-diol present in most AZAs can be oxidatively cleaved with periodate, and this reaction has been used as a tool in structure elucidation of novel AZAs (Kilcoyne et al., 2014a, 2014b, 2018; Rehmann et al., 2008). However, AZAs containing fatty acid esters on C-20 or C-21 would not be expected to undergo the cleavage reaction with periodate, given that there would no longer be a 1,2-diol present at C-20 and C-21. The semi-synthetic 3-*O*-palmitoylAZA4 reacted completely with periodate within 2 h, providing additional confirmation that esterification took place on the C-3 hydroxy, and not on the C-20 hydroxy. The AZA esters in the Bruckless extract were also cleaved by periodate, confirming that the esters present *in vivo* formed via reaction with either the C-3 or C-23 hydroxy group, while the C-20 and C-21 hydroxy groups had not been esterified, possibly due to steric hindrance or enzyme specificity (Fig. S7).

### 3.4 Presence of AZA esters in CRMs and other tissues

Additional mussel tissues and reference materials were evaluated for the presence of AZA fatty acid esters . As noted, the Bruckless mussel tissues analyzed in this study were used in the preparation of two CRMs (CRM-AZA-Mus and CRM-FDMT1). Consequently, the presence of AZA fatty acid esters in these reference materials provides positively-characterized materials that are available for use in other studies. 3-*O*-palmitoylAZA4 and 3-*O*-oleoylAZA4 (the 16:0 and 18:1 esters, respectively) were detected in all samples evaluated. CRM-FDMT1 and CRM-AZA-Mus (McCarron et al., 2015, 2017) contained trace levels of additional AZA4 fatty acid esters (Table S2), primarily those that had a peak area >10% relative to 3-*O*-palmitoylAZA4 in the Bruckless sample (Table 1). The content of 3-*O*-palmitoylAZA4 was estimated in the tissue extracts based on the peak areas of its [M+H]^+^ ion in full scan mode relative to that of AZA4 in the certified tissue of CRM-FDMT1 assuming the same molar response factor, and is summarized in Table 2. These semi-quantitative estimates indicate approximate levels ranging from 0.005 to 0.03 mg/kg of 3-*O*-palmitoylAZA4 in fresh whole mussel homogenates, with higher concentrations in the freeze-dried and HP tissues at 0.15 mg/kg. This represents 0.2–2.5% of the total free AZA1–4 concentrations by weight in the tissues. The mussels from Killary Harbour that were implicated in the original AZA poisoning event (Satake et al., 1998) contained only 3-*O-*palmitoyl- and 3-*O*-oleoylAZA4 at trace levels, while the mussel sample from Gouladoo, Bantry Bay, contained a higher content of AZA4 3-*O*-acyl esters including 10 different acyl moieties, similar to those observed in high abundance in the Bruckless HP, as well as 3-*O*-palmitoylAZA-9.

**Table 2:** Estimated concentrations of AZA1–4 and 3-*O*-palmitoylAZA4 (i.e. 16:0 esters) in several AZA-incurred materials assuming equivalent molar response to AZA4 in CRM-FDMT1.

Tissue Sample	Concentration (mg/kg)				
	AZA1	AZA2	AZA3	AZA4	16:0 AZA4^a^
FDMT1^b^	4.10 ± 0.40	1.13 ± 0.10	0.96 ± 0.10	0.42 ± 0.01	0.15 ± 0.01
CRM-AZA-mus^b^	1.16 ± 0.10	0.27 ± 0.02	0.21 ± 0.02	0.17 ± 0.01	0.02 ± 0.01
Bruckless HP (2005)	2.85 ± 0.11	1.36 ± 0.06	1.34 ± 0.04	0.36 ± 0.02	0.15 ± 0.01
Gouladoo (2012)	3.64 ± 0.01	1.26 ± 0.04	0.60 ± 0.02	0.15 ± 0.01	0.03 ± 0.01
Killary (1995)	1.13 ± 0.12	0.39 ± 0.04	0.17 ± 0.02	0.07 ± 0.01	0.005 ± 0.001

a Calculated as AZA4 equivalents.

b AZA1–3 concentrations and uncertainty based on certified values.

Relative toxicities of AZAs based on *in vitro* assays have shown that methylation on C-22 increases toxicity, while hydroxylation on C-3 or C-23 reduces toxicity (Kilcoyne et al., 2018). While the okadaic acid and dinophysistoxin acyl esters have lower toxicities relative to their free toxins, there is limited information available on the toxicity of many acyl ester metabolites of algal toxins in shellfish (Torgersen et al., 2005). The AZA fatty acid esters detected in Atlantic European mussels in this work were of low relative abundance compared to the major free AZAs, and were formed for C-3 or C-23 hydroxylated AZAs with only trace levels of esters for AZAs with methylation at C-22, suggesting they are of reduced toxicological significance. However, further work should investigate ester formation for other AZA analogues, as well as AZA ester distribution across mussel tissues and variation across a larger sample set to determine species and location differences. It should also be considered that AZA esters may undergo hydrolysis during digestion, which presents a risk of exposure to higher levels of the toxins than predicted based on the analysis of free toxins. Future investigations will require optimization of the semi-synthesis reaction conditions for a broader range of AZAs in order to improve quantitative measurements of AZA fatty acid esters and to provide toxicological information.

In mussel feeding studies with *A. spinosum,* AZA1–2 appeared within 6 h and remained at relatively constant concentrations, while the concentration of total metabolites continued to increase for the duration of the feeding experiments (Jauffrais et al., 2012a, 2012b; Salas et al., 2011). Acylation of okadaic acid has been shown to be involved in the depuration of these toxins from shellfish (Rossignoli et al., 2011) and may have a similar function with AZAs. Given that during shellfish feeding with *Azadinium* spp., AZA accumulation appears to stabilize, there may be additional biotransformations such as *O*-acylation that could contribute to an increase in total AZAs over time. Future studies evaluating the accumulation, transformation and depuration of AZAs in shellfish should consider the significance of additional metabolites such as fatty acid esters.

## 4 Conclusions

The presence of AZA fatty acid esters was confirmed in mussel tissues using LC-HRMS/MS and chemical reactions. These hydrophobic metabolites elute after previously reported AZAs, but share common MS/MS fragmentation characteristics, with the majority of esters observed being formed from AZA4 or AZA9 with trace levels formed from AZA5, AZA7 and AZA13. The primary fatty acid involved in esterification was palmitic acid (16:0), which was confirmed based on semi-synthesis of 3-*O*-palmitoylAZA4. The discovery of these acyl esters highlights an additional metabolic pathway for AZAs in shellfish, and shellfish feeding studies would be important to study their accumulation, transformation and depuration kinetics. The methodologies employed here can be used to evaluate a broader range of AZA-incurred materials to compare ester profiles and the variation in esterification across different shellfish species.

## CRediT authorship contribution statement

**Elizabeth M. Mudge:** Methodology, Investigation, Writing - original draft, Writing - review & editing. **Christopher O. Miles:** Writing - review & editing. **William R. Hardstaff:** Investigation, Writing - review & editing. **Pearse McCarron:** Conceptualization, Methodology, Investigation, Writing - review & editing.

## Declaration of competing interest

The authors declare no conflicts of interest.
