# Supplementary material for: Fatty acid esters of azaspiracids identified in mussels (Mytilus edulis) using liquid chromatography-high resolution mass spectrometry
Source: Toxicon X. 2020 Sep 28;8:100059. doi: 10.1016/j.toxcx.2020.100059 (PMC7549145; doi:10.1016/j.toxcx.2020.100059)
Supplement: Multimedia component 1 [file mmc1.docx]

**Fatty Acid Esters of Azaspiracids Identified in Mussels (*Mytilus edulis*) using Liquid Chromatography-High Resolution Mass Spectrometry**

Elizabeth M. Mudge, Christopher O. Miles, William R. Hardstaff, Pearse McCarron*

Biotoxin Metrology, National Research Council Canada, 1411 Oxford St., Halifax, Nova Scotia, B3H 3Z1, Canada

*Corresponding Author:

Phone: +1 (902) 426-6182

Email: [Pearse.McCarron@nrc-cnrc.gc.ca](mailto:Pearse.McCarron@nrc-cnrc.gc.ca)

**Supplementary Data**

**Table S1.** Inclusion list for DIA acquisition with the mass centers for each of the 15 acquisition windows with a mass width of *m*/*z* 39, and the stepped collision energy (NCE).

| Mass (*m*/*z*) | Polarity | NCE |
| --- | --- | --- |
| 668.0000 | Positive | 35, 65 |
| 705.0000 | Positive | 35, 65 |
| 742.0000 | Positive | 35, 65 |
| 778.0000 | Positive | 35, 65 |
| 815.0000 | Positive | 35, 65 |
| 852.0000 | Positive | 35, 65 |
| 888.0000 | Positive | 35, 65 |
| 925.0000 | Positive | 35, 65 |
| 962.0000 | Positive | 35, 65 |
| 998.0000 | Positive | 35, 65 |
| 1035.0000 | Positive | 35, 65 |
| 1072.0000 | Positive | 35, 65 |
| 1108.0000 | Positive | 35, 65 |
| 1145.0000 | Positive | 35, 65 |
| 1182.0000 | Positive | 35, 65 |

**Table S2.** Summary of 3-*O*-acyl AZA esters detected in whole mussel tissues. (+) detected, (-) not detected.

| Ester | CRM-FDMT1 | CRM-AZA-Mus | Killary | Gouladoo |
| --- | --- | --- | --- | --- |
| 14:0 AZA4 | + | + | - | + |
| 15:0 AZA4 | + | + | - | + |
| 16:1 AZA4 | + | + | - | + |
| 16:0 AZA4 | + | + | + | + |
| 17:1 AZA4 | + | + | - | + |
| 17:0 AZA4 | + | + | - | + |
| 18:1 AZA4 | + | + | + | + |
| 18:0 AZA4 | - | - | - | + |
| 20:5 AZA4 | - | - | - | + |
| 20:2 AZA4 | + | + | - | - |
| 22:6 AZA4 | - | + | - | + |
| 16:0 AZA9 | - | - | - | + |


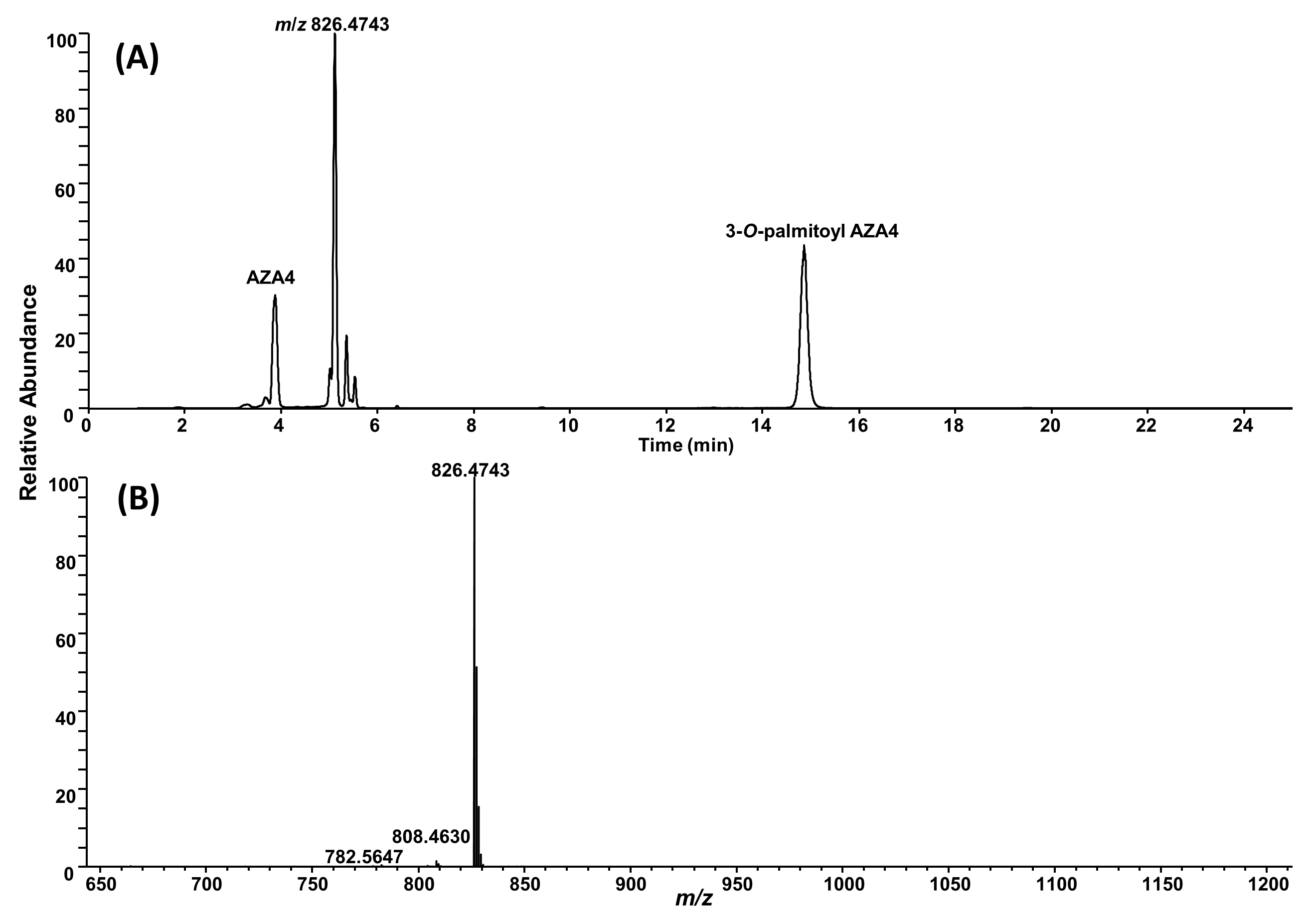


**Figure S1.** (A) Extracted ion chromatogram of AZA4, the product at *m*/z 826.4743 and 3-*O*-palmitoyl AZA4 from semi-synthesis of 3-*O*-palmitoyl (16:0) AZA-4. (B) Full scan MS spectrum of the product with [M+H]^+^ of *m*/*z* 826.4743, suspected to be due to 2,3-dehydroAZA4 (C_46_H_68_O_12_N^+^, Δ 0.8 ppm) based on periodate cleavage of the 20,21-diol (data not shown).


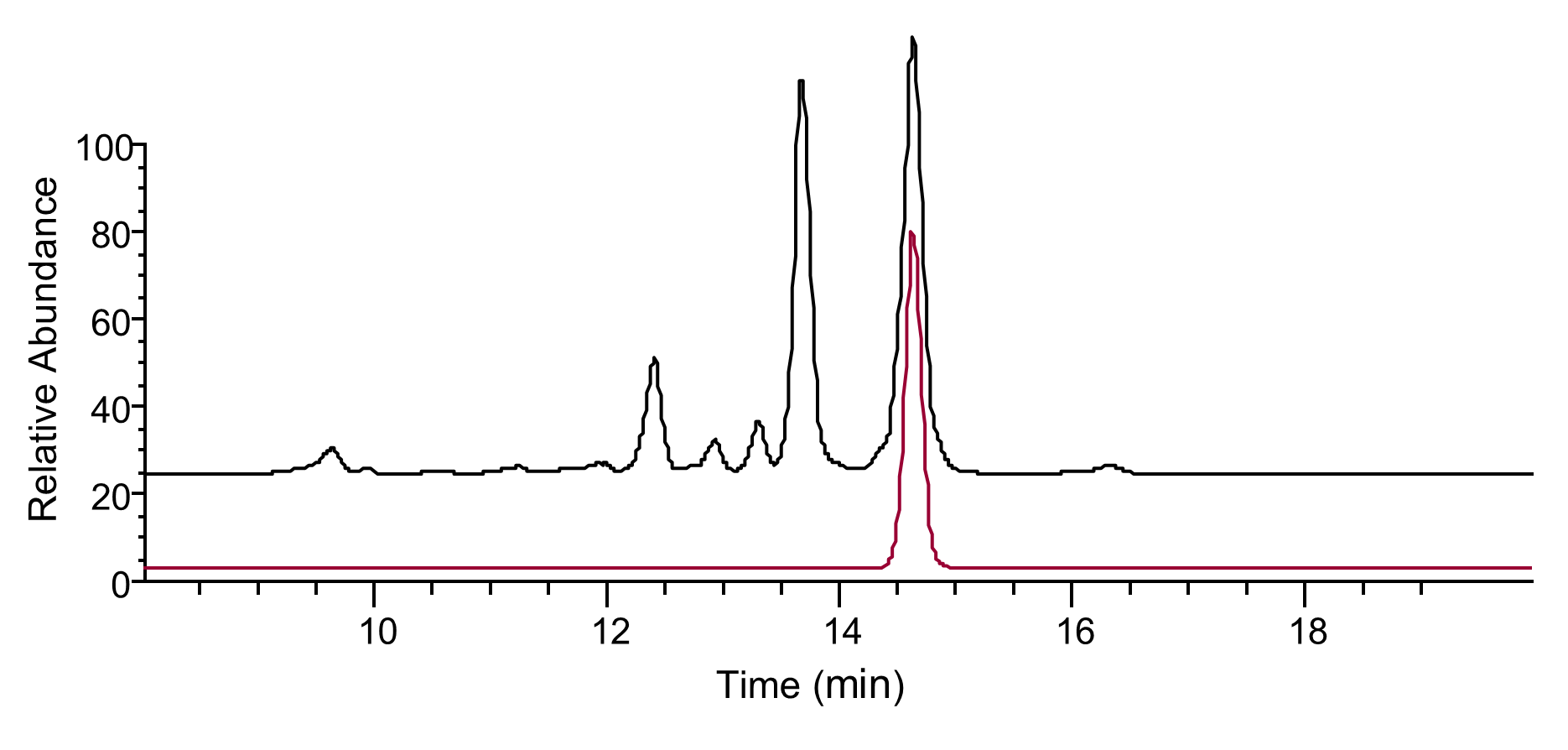


**Figure S2.** Extracted ion chromatogram of *m*/*z* 1082.7138 (±5 ppm) of the Bruckless HP tissue (black) and the semi-synthesized 3-*O*-palmitoylAZA4 (red).


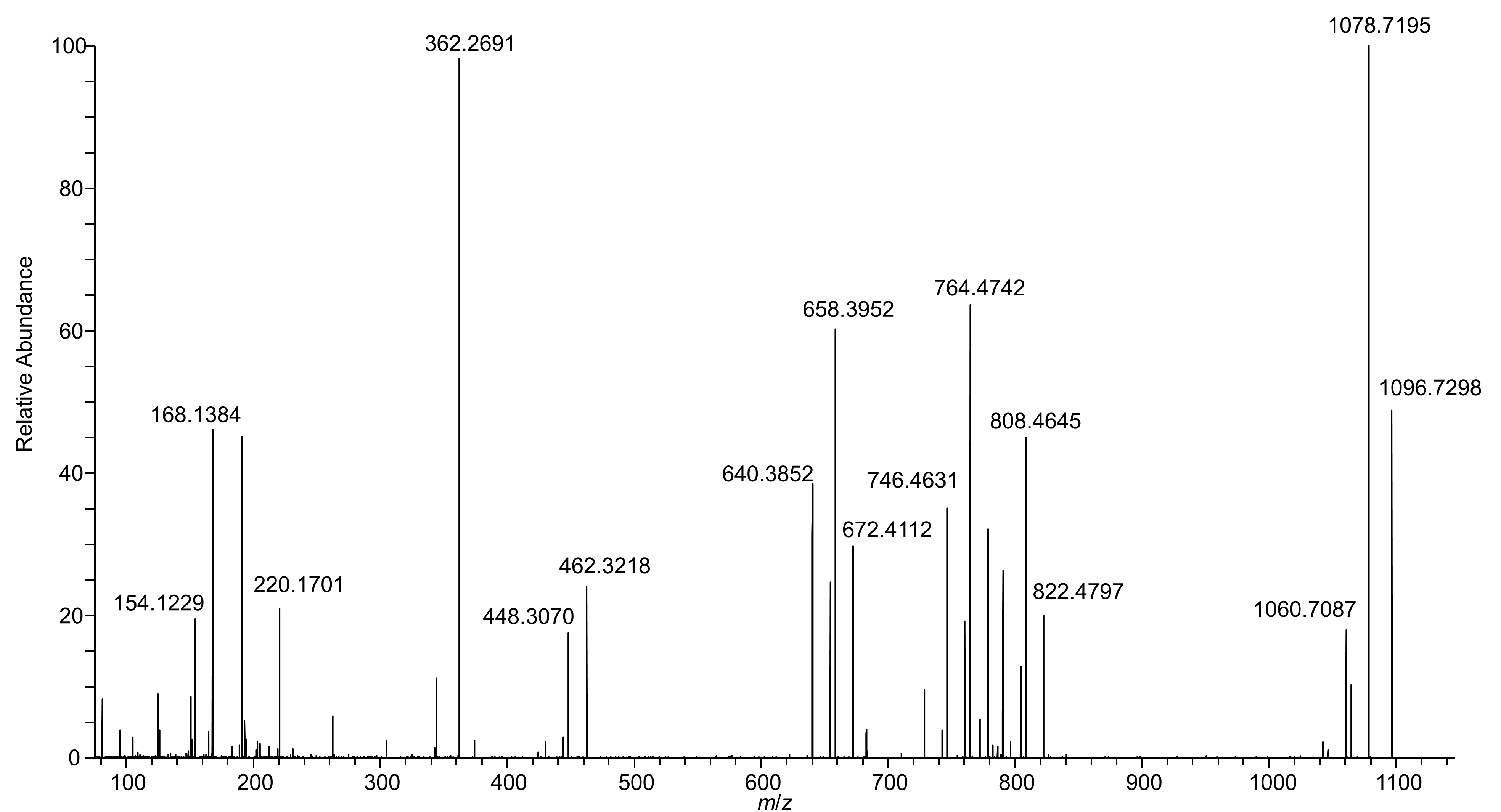


**Figure S3.** Product ion spectra of 3-*O*-pamitoylAZA7 co-eluting with 3-*O*-margaroylAZA4 in the concentrated 7:3 EtOAc:MeOH silica gel fraction of the hepatopancreas tissue. The vertical scale from *m*/*z* 100-950 was expanded 20× to highlight diagnostic ions.


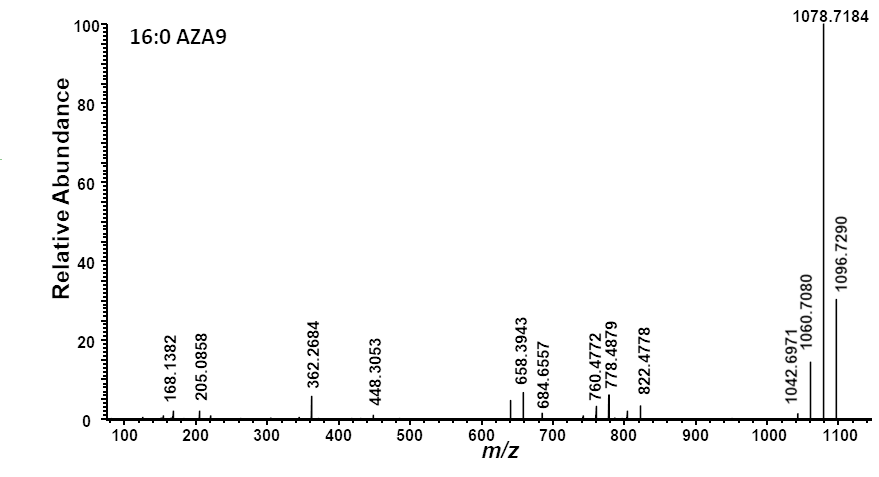


**Figure S4.** Product ion spectrum of semi-synthetic 3-*O*-palmitoylAZA9, which was confirmed to be present in the HP tissue.


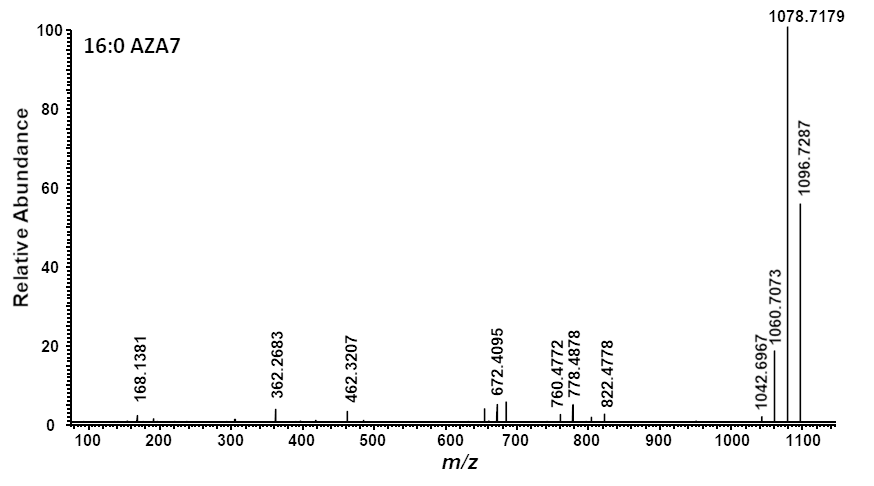


**Figure S5.** Product ion spectrum of the semi-synthetic 3-*O*-palmitoylAZA7.


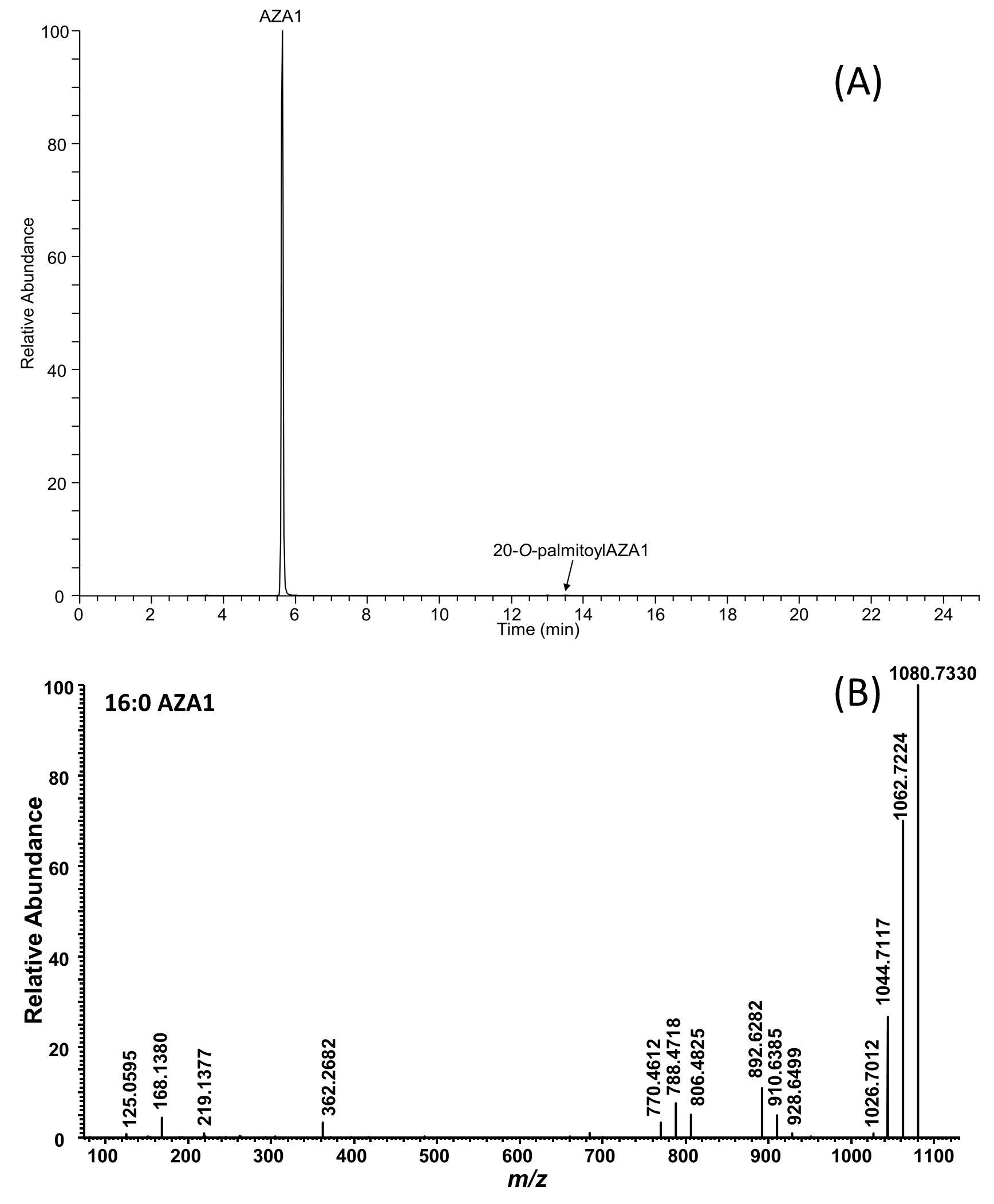


**Figure S6.** (A) Extracted ion chromatogram of AZA1 (*m*/*z* 842.5049) and of the semi-synthetic 16:0 ester of AZA1 (presumed to be 20-*O*-palmitoylAZA1, *m*/*z* 1080.7345) (B) Product ion spectrum of the semi-synthesized 20-*O*-palmitoylAZA1), which was not detected in the HP tissue.


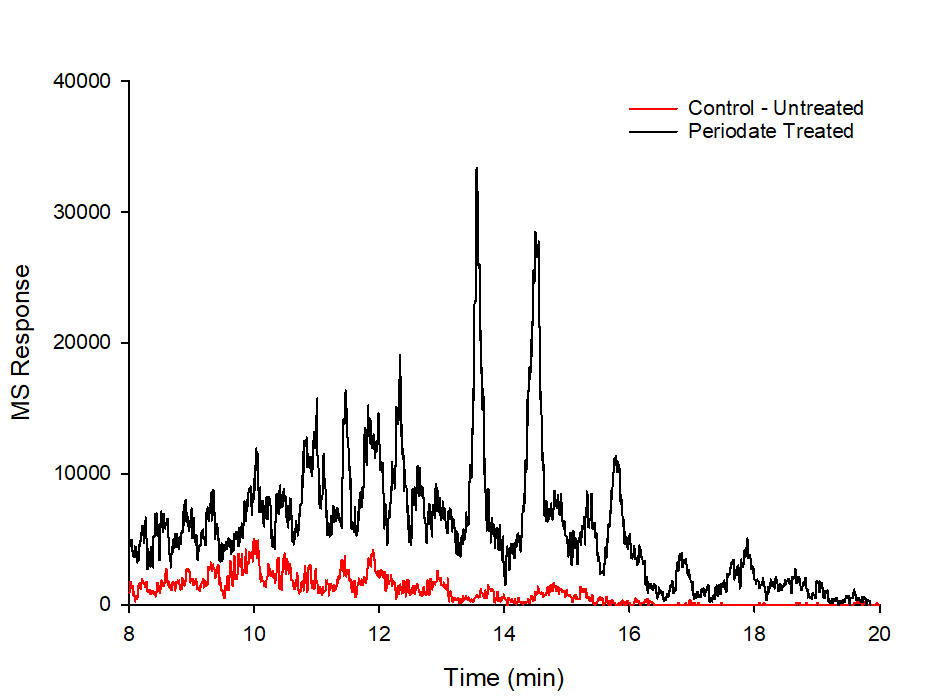


**Figure S7.** Extracted ion chromatogram of the product ions at *m*/*z* 362.2690 and 168.1381 from the mass range of *m*/*z* 650 to 1200 using DIA acquisition in the HP tissue in a control extract (black) and 2 h after treatment with sodium periodate.
